# Unusual Coincidence: Concurrent Cast Nephropathy and Lymphoma Infiltration in an Influenza A-Associated Acute Kidney Injury

**DOI:** 10.1155/2024/5524746

**Published:** 2024-03-01

**Authors:** Wan-Ching Lee, Chun-Kuang Tsai, Szu-Yuan Li

**Affiliations:** ^1^Division of Nephrology, Department of Internal Medicine, Taipei Veterans General Hospital, Taipei 11217, Taiwan; ^2^Division of Hematology, Department of Internal Medicine, Taipei Veterans General Hospital, Taipei 11217, Taiwan; ^3^School of Medicine, National Yang Ming Chiao Tung University, Taipei 11217, Taiwan

## Abstract

Acute kidney injury (AKI) poses a substantial challenge in the management of lymphoma patients and is frequently associated with diverse causative factors. Herein, we report an illustrative case involving a 47-year-old male with influenza A infection who developed severe AKI, which was incongruent with his medical history. Laboratory investigations disclosed aberrant immunoglobulin levels and urinary protein excretion, prompting further evaluation. A renal biopsy revealed the presence of infiltrating lymphoid cells and cast nephropathy, raising suspicion of an underlying hematological disorder. A comprehensive diagnostic workup, including positron emission tomography imaging and bone marrow biopsy, culminated in the definitive diagnosis of splenic marginal zone lymphoma. This case highlights the crucial significance of including lymphoma-associated kidney disorders in the evaluation of unexplained AKI, particularly when encountering unconventional clinical and laboratory results. Swift and precise intervention is of utmost importance in attaining positive results in these rare and complex clinical situations. This study underscores the persistent concern of AKI in lymphoma patients, with lymphocytic infiltration and cast nephropathy as notable elements contributing to the intricate nature of this condition.

## 1. Introduction

In patients with lymphoma, the development of kidney injury is a matter of substantial concern, impacting approximately one-third of cases [1]. It is often associated with a diverse array of factors such as lymphoid cell infiltration, sepsis, metabolic disruptions, and monoclonal immunoglobulin deposition [2]. Despite its prevalence, kidney injury frequently eludes early diagnosis in lymphoma patients due to the insidious and slow progression of lymphocytic infiltration. Symptoms may include flank pain and blood in the urine. Light-chain cast nephropathy (LCCN) arises from an excess of monoclonal free light chains, leading to inflammation and scarring in the proximal tubules of the kidneys. It is among the most frequent kidney complications in multiple myeloma [3]. It is vital to promptly and effectively lower the free light chain levels for the treatment of cast nephropathy [4]. This case report highlights a patient diagnosed with indolent B-cell lymphoma, whose renal biopsy uncovered the presence of both cast nephropathy and lymphoid cell infiltration.

## 2. Case Report

A 47-year-old male, confirmed with influenza A via rapid antigen test, presents with an acute sudden onset of symptoms. He developed fever, general malaise, a productive cough, and decreased appetite 10 days ago after contracting influenza A. Following five days of oseltamivir treatment, he experienced worsening shortness of breath and reduced urine output (<100 ml per day). Over the subsequent week, he gained 6 kilograms in weight, prompting him to seek medical assistance. The patient's medical history includes hypertension and coronary artery disease. Asymptomatic chronic leukocytosis without a definite diagnosis was noted for six months, which is distinct from his current presentation. He is currently taking amlodipine and bisoprolol for his hypertension, with a blood pressure reading of 142/99 mmHg. Physical examination revealed multiple palpable lymph nodes in the bilateral neck and lower limb edema, with no signs of petechiae or purpura.

Laboratory findings showed a white blood cell count of 72,860/uL with 73% lymphocyte, hemoglobin at 9.7 g/dL, and platelet count of 192,000/uL. Small to medium size lymphocytes with irregular nuclear around 11% of leukocytes were found in the peripheral blood. We also found a CRP of 3.43 mg/dL, serum albumin of 3.3 g/dL, sodium at 123 mEq/L, serum potassium at 3.8 mEq/L, calcium level at 8.6 mg/dL, and negative results for ANA and ANCA. His BUN was elevated at 53.6 mg/dL, with a creatinine level of 13.85 mg/dL (baseline was 0.71 mg/dL four months ago). Serum immunoglobulin G (IgG) levels were elevated at 3435 mg/dL, with a reversed A/G ratio of 0.6, and the serum-free light-chain kappa/lambda ratio was 308.26. Urinalysis indicated 3+ protein, trace blood, 1+ leukocyte esterase, and 50–99 white blood cells per high power field (WBC/HPF). Urine sediment examination revealed no crystals or casts. The urine protein-to-creatinine ratio was 4.6. Kidney ultrasound indicated a kidney size of 11.5 cm without hydronephrosis. An excisional biopsy of the left neck lymph node showed benign fibroadipose tissue. The patient received hemodialysis to manage refractory pulmonary edema and electrolyte imbalances.

**Figure 1:**
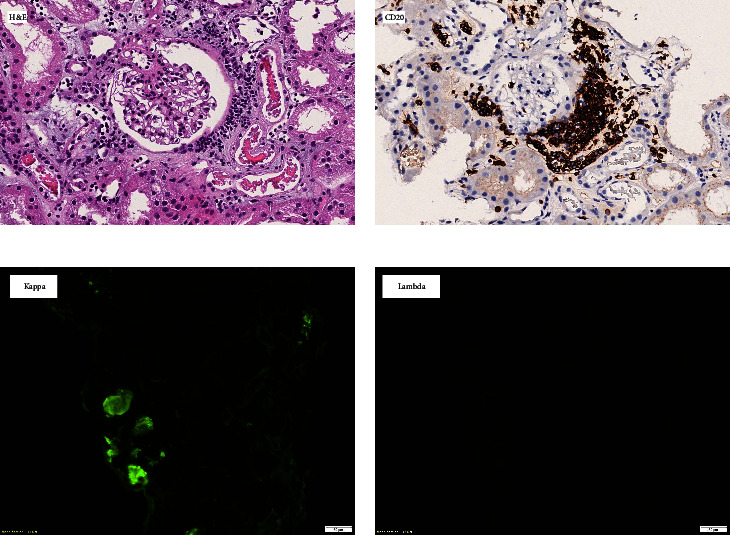
Kidney biopsy with (a) hematoxylin and eosin stain showing atypical tubular casts with sharp-edged or fractured appearance with atypical lymphoid cells around glomerulus. (b) Those atypical lymphoid cells were CD20 positive. (c and d) By immunofluorescence, these atypical casts are strongly positive for kappa staining but nonreactive for lambda staining.

**Figure 2:**
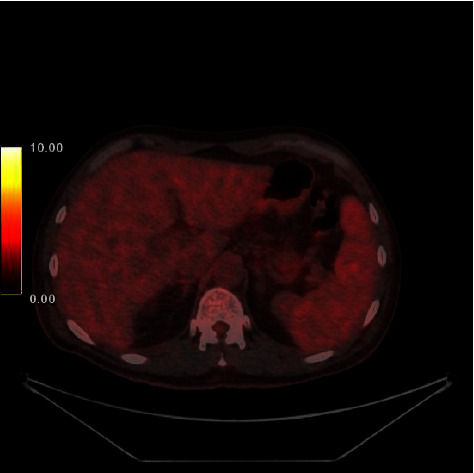
Positron emission tomography (PET) scan displayed mild and diffusely increased uptake (score 4, uptake slightly to moderately higher than liver) at the spleen with splenomegaly, compatible with lymphoma involvement.

**Figure 3:**
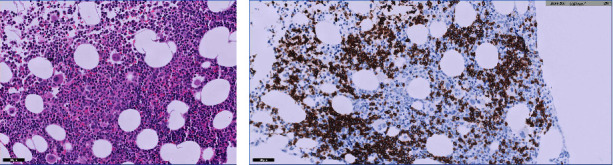
Bone marrow biopsy with (a) hematoxylin and eosin stain showing small clusters of small lymphoid cells with interstitial infiltration of about 40% and (b) the neoplastic cells are positive for CD20, compatible with low-grade mature B-cell neoplasm.

After completing three sessions of hemodialysis, the patient underwent a kidney biopsy. The biopsy findings showed the presence of atypical tubular casts with fragmented appearances. Notably, there was an infiltration of abnormal lymphoid cells, which were found to be responsive to CD20 and unresponsive to CD3 and cyclin D1, as shown in Figure 1. A positron emission tomography (PET) scan displayed slight and widespread uptake in the spleen, coinciding with splenomegaly (Figure 2). Results from a bone marrow biopsy indicated the presence of a mature low-grade B-cell lymphoma. Immunohistochemical staining was positive for CD20 but negative for CD3, CD5, CD10, CD23, cyclin D1, LEF1, CD200, FcRL4, and IgD/IgM. MYD88 mutation test was negative for L265P (Figure 3). Consequently, a diagnosis of splenic marginal zone lymphoma was confirmed. Following treatment with rituximab and bendamustine, the patient's blood leukocyte count normalized. With the patient demonstrating normal urine output and improved kidney function, renal replacement therapy was discontinued within a week. Upon discharge, the patient's serum creatinine level had returned to 1.8 mg/dL.

## 3. Discussion

In lymphoma patients, acute kidney injury (AKI) is a notable concern impacting 31.8% of cases and correlating with heightened mortality and prolonged hospitalization [1]. Various factors, such as lymphoid cell infiltration, sepsis, metabolic disturbances, and monoclonal immunoglobulin deposition, including tumor lysis syndromes, can cause AKI [2]. Among the spectrum of kidney disorders associated with lymphoma, lymphocytic infiltration emerges as the most common. An autopsy study discovered parenchymal invasion in 34% of lymphoma cases, yet only one-third was diagnosed during their lifetime [5]. The underdiagnosis may be attributed to the subtle and gradual progression of lymphocytic infiltration, with most cases not necessitating dialysis. Possible symptoms include nonspecific flank pain, hematuria, a detectable mass, hypertension, and proteinuria. The underlying mechanism might involve an abundance of lymphoid cells obstructing renal tubules, although kidney damage of this kind typically ameliorates within 1–4 weeks following chemotherapy [6, 7].

In our specific case, the patient exhibited acute kidney injury requiring dialysis. Upon comprehensive review of the patient's medical history, an undiagnosed case of small B-cell neoplasm was unveiled. Laboratory findings revealed evidence of light chain production. A kidney biopsy confirmed the diagnosis of light-chain cast nephropathy (kappa type) accompanied by the infiltration of atypical lymphoid cells. Subsequent investigations, including a bone marrow biopsy and a PET scan, culminated in the definitive diagnosis of splenic marginal zone lymphoma (SMZL). SMZL is a rare, indolent B-cell lymphoma affecting the spleen, bone marrow, and often the blood, accounting for 0.6% of non-Hodgkin lymphoma (NHL) cases [8]. A transition to large B-cell lymphoma occurs in 10–20% of patients [9]. Cases of SMZL with monoclonal gammopathy are exceedingly rare [10]. The case of our patient potentially represents the first instance of SMZL complicated by cast nephropathy and lymphoma infiltration.

Light-chain cast nephropathy (LCCN) arises due to an excess of monoclonal free light chains (FLCs) in renal tubules. Several precipitating factors may contribute to its onset, such as volume depletion, hypercalcemia, or infections [3]. The casts display a fragmented appearance, which is formed by the combination of Tamm–Horsfall protein and monoclonal light chains. These casts can lead to tubular obstruction and direct toxicity to the tubules. They exhibit eosinophilia when subjected to H & E staining. Their geometric shapes may vary, and they may be encircled by epithelial, giant, or inflammatory cells, sometimes accompanied by interstitial inflammation. On immunofluorescence, the casts exhibit a restriction to either kappa or lambda light chains.

In conclusion, AKI in lymphoma patients represents a notable concern given its multifactorial etiology. Lymphocytic infiltration, a common but less frequent biopsy-proven cause, may lead to delayed diagnosis. Our report highlights a distinctive case of SMZL with cast nephropathy and lymphoma infiltration. Prompt initiation of chemoimmunotherapy proved instrumental in restoring renal function in this instance.

## Data Availability

The text includes deidentified data supporting the conclusions presented in this case report.
